# The Anti-Leukemia Effect of Ascorbic Acid: From the Pro-Oxidant Potential to the Epigenetic Role in Acute Myeloid Leukemia

**DOI:** 10.3389/fcell.2022.930205

**Published:** 2022-07-22

**Authors:** S. Travaglini, C. Gurnari, S. Antonelli, G. Silvestrini, N. I. Noguera, T. Ottone, M. T. Voso

**Affiliations:** ^1^ Department of Biomedicine and Prevention, University of Rome Tor Vergata, Rome, Italy; ^2^ Department of Translational Hematology and Oncology Research, Taussig Cancer Institute, Cleveland Clinic, Cleveland, OH, United States; ^3^ Neuro-Oncohematology Unit, IRCCS Fondazione Santa Lucia, Rome, Italy

**Keywords:** acute myeloid leukemia, ascorbic acid, epigenetic regulation, oxidative stress, vitamin C

## Abstract

Data derived from high-throughput sequencing technologies have allowed a deeper understanding of the molecular landscape of Acute Myeloid Leukemia (AML), paving the way for the development of novel therapeutic options, with a higher efficacy and a lower toxicity than conventional chemotherapy. In the antileukemia drug development scenario, ascorbic acid, a natural compound also known as Vitamin C, has emerged for its potential anti-proliferative and pro-apoptotic activities on leukemic cells. However, the role of ascorbic acid (vitamin C) in the treatment of AML has been debated for decades. Mechanistic insight into its role in many biological processes and, especially, in epigenetic regulation has provided the rationale for the use of this agent as a novel anti-leukemia therapy in AML. Acting as a co-factor for 2-oxoglutarate-dependent dioxygenases (2-OGDDs), ascorbic acid is involved in the epigenetic regulations through the control of TET (ten-eleven translocation) enzymes, epigenetic master regulators with a critical role in aberrant hematopoiesis and leukemogenesis. In line with this discovery, great interest has been emerging for the clinical testing of this drug targeting leukemia epigenome. Besides its role in epigenetics, ascorbic acid is also a pivotal regulator of many physiological processes in human, particularly in the antioxidant cellular response, being able to scavenge reactive oxygen species (ROS) to prevent DNA damage and other effects involved in cancer transformation. Thus, for this wide spectrum of biological activities, ascorbic acid possesses some pharmacologic properties attractive for anti-leukemia therapy. The present review outlines the evidence and mechanism of ascorbic acid in leukemogenesis and its therapeutic potential in AML. With the growing evidence derived from the literature on situations in which the use of ascorbate may be beneficial *in vitro* and *in vivo*, we will finally discuss how these insights could be included into the rational design of future clinical trials.

## Introduction

Acute myeloid leukemia (AML) is a heterogeneous clonal disorder characterized by the uncontrolled proliferation of undifferentiated myeloid progenitor cells in the bone marrow and peripheral blood. Advances in DNA sequencing technologies have provided a detailed knowledge of the molecular landscape of AML, with a better understanding of the disease pathogenesis and prognosis (The Cancer Genome Atlas Research Network, 2013; Papaemmanuil et al., 2016; Awada et al., 2021). Despite high heterogeneity, the spectrum of genetic alterations have highlighted the presence of recurrent mutations in genes encoding epigenetic regulators (Gallipoli et al., 2015), including DNA methyltransferase 3A (DNMT3A), Ten-eleven-translocation 2 (TET2), Wilms’ tumor 1 (WT1), and isocitrate dehydrogenase 1 and 2 (IDH1/2) (Kunimoto and Nakajima, 2017). The study of the molecular architecture of such cases unveiled that alterations of these genes often represent founding events in the clonal hierarchy, suggesting their essential role in early phase of leukemia ontogenesis (Papaemmanuil et al., 2013; Hirsch et al., 2018; Yoshimi et al., 2019).

Epigenetic mechanisms play a major role in normal and, particularly, in malignant hematopoiesis, where the presence of recurrent alterations in transcription factors and chromatin regulators are able to drive hematopoietic malignancies (Hu and Shilatifard, 2016; Fennell et al., 2019). Epigenetic modifiers are a large and varied group of proteins involved in the modification of DNA at cytosine residues and post-translational acetylation and methylation of histones, whose aberrations lead to dysregulation of critical genes that control cell growth, differentiation and apoptosis, all important mechanisms in the pathogenesis of AML (Hou and Tien, 2016; Pastore and Levine, 2016). In contrast to genetic changes, epigenetic modifications refer to changes in gene expression, and are frequently reversible, providing the opportunities for targeted treatment (Wouters and Delwel, 2016).

A wide range of therapeutic strategies that target epigenetic alterations in AML have been successfully tested in preclinical studies and several drugs have already gained approval for clinical use, with the final goal to reverse epigenetic dysfunctions (Bewersdorf et al., 2019). Since these discoveries, a number of selective inhibitors of mutant FLT3, IDH1 and IDH2 have been developed and are now approved by the Food and Drug Administration, and drugs that target the potential epigenetic writers (DOT1L, PRMT5), readers (BRD2/3/4) and erasers (HDAC, LSD1) are currently under investigation (Das et al., 2021). However, the therapeutic efficacy of epigenetic drugs as single agents seems to be limited (Bewersdorf et al., 2019).

Ascorbic acid, also known as vitamin C or ascorbate, at physiological levels potentiates the effects of hypomethylating agents (HMA) by both causing DNA demethylation through active and passive mechanisms and selectively killing tumor cells (Ruiz et al., 2015; Liu et al., 2016; Gillberg et al., 2018). Indeed, ascorbic acid is an essential factor for epigenetic regulation and many studies reported that it also displays a pro-oxidant activity when used at high concentrations, in particular on various type of cancer cells, suggesting it as an emerging epigenetic therapy (Chen et al., 2005; Chen et al., 2008).

Besides its role in epigenetics, ascorbic acid is a pivotal regulator of many physiological processes in humans (Giansanti et al., 2021), including cellular immune responses (Ströhle et al., 2011), and represents an antioxidant molecule involved in the reactive oxygen species (ROS) scavenging that prevent oxidative DNA damage and other effects, which may lead to cancer transformation.

In this review, we provide an overview of the role of ascorbate as an essential factor for epigenetic regulation, highlighting its anti-leukemic mechanisms of action, currently under investigation in the treatment of AML.

## Ascorbic Acid: Structure, Biosynthesis and Uptake

Ascorbic acid represents a wide spectrum antioxidant and an essential nutrient for humans. This vitamin exists in two redox states, ascorbate, the reduced active form, and dehydroascorbic acid (DHA), its oxidized form. At physiological conditions, ascorbic acid loses a proton to form the ascorbate anion (AscH−), the predominant ionic form of vitamin C, that can be oxidized by losing two protons, giving rise first to ascorbate radical (Asc^−^) and then to a fully oxidized DHA (Ferrada et al., 2021).

Unlike most mammals, humans are unable to synthesize ascorbic acid from oxidated glucose, missing the gulonolactone (L-) oxidase (GULO), a key enzyme involved in the catalysis of the last enzymatic step in ascorbate synthesis, and are thereby dependent on vitamin C intake from the diet (Du et al., 2012). The dietary daily requirement of vitamin C is 75–90 mg/day, which is usually taken under the form of both ascorbate and DHA (Valdés, 2006; Testa et al., 2021). Indeed, even if DHA levels are extremely low under physiological conditions (Du et al., 2012), it is rapidly recycled back to ascorbate by DHA reductase, using glutathione (GSH) as a reducing agent (Mandl et al., 2009). This recycling process occurs intracellularly, where vitamin C is involved in several biological processes. Thus, its exogenous uptake is attained through the presence of specific membrane transporters, which determine the distribution of this molecule between extra- and intra- cellular compartments. Both ascorbic acid and DHA can be transported across the plasma membrane, although by distinct carriers (Savini et al., 2008). Under physiologic conditions, vitamin C is mainly present in plasma as ascorbate and is actively absorbed in the gastrointestinal tract, particularly at the level of the enterocytes of the small intestine (May, 2011; Du et al., 2012).

**FIGURE 1:**
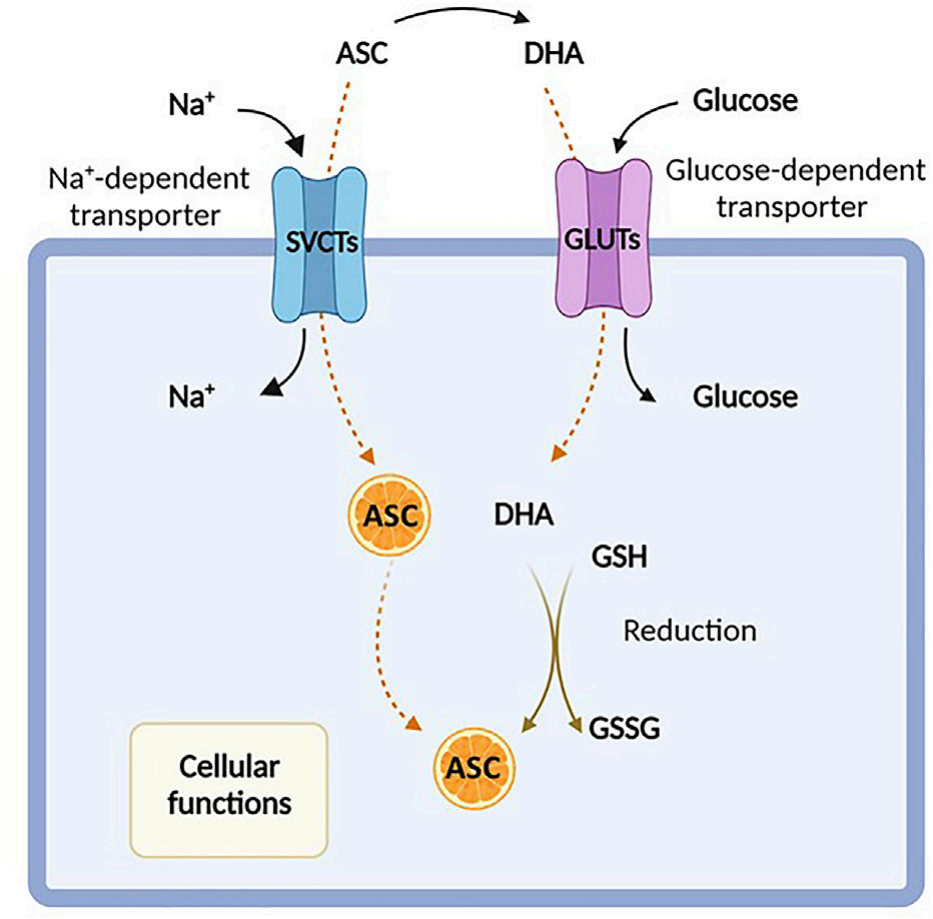
Ascorbic acid uptake. Ascorbic acid, also known as Vitamin C, enters the cell either in its reduced form (ascorbate, ASC) by sodium-dependent vitamin C transporters (SVCTs) or in its oxidized form (dehydroascorbate, DHA) *via* facilitative glucose transporters (GLUTs). In the cytosol, DHA is rapidly reduced back to ASC in the presence of glutathione (GSH). Created with Biorender.com.

Ascorbate is transported into mammalians cells in an energy-dependent fashion, requiring two types of sodium-dependent transporters, SVCT1 and SVCT2, encoded by *SLC23A1* and *SLC23A2* genes, respectively, which show distinct tissue distributions (Tsukaguchi et al., 1999; Sotiriou et al., 2002; May, 2011). Conversely, DHA can be internalized by facilitative hexose transporters, particularly via GLUT1 and GLUT3 (Rumsey et al., 1997; Rumsey et al., 1999), encoded by the *SLC2A1* and *SLC2A3* genes, and then converted back to ascorbic acid (Savini et al., 2008) (Figure 1). As mentioned above, DHA is present at extremely low concentration in the blood of healthy subjects and at much higher levels in the intestine, and its uptake is inhibited by excess glucose (Vera et al., 1993; Malo and Wilson, 2000; Corpe et al., 2013; Lykkesfeldt and Tveden-Nyborg, 2019). However, the intracellular transport of DHA by GLUT transporters is not usually considered the principal route of uptake, even if the impact of ascorbate and/or DHA to cellular transport *in vivo* is not fully elucidated (Rumsey and Levine, 1998).

Vitamin C uptake and its whole body distribution is essential for many biochemical processes, some of them also influencing tumor growth and spread (Wohlrab et al., 2017). Indeed, the ascorbate concentration in plasma and tissues represent one of the main risk factor for cancer incidence (Linowiecka et al., 2020). Several studies reported also that cancer patients are more likely to experience vitamin C deficiency due to a variety of factors such as decreased oral intake, inflammation, infection, and disease phases, particularly following chemotherapy and hematopoietic stem cells (HSCs) transplantation, resulting in shorter survival (Caraballoso et al., 2003; Mayland et al., 2005; Nannya et al., 2014; Wilson et al., 2014; Rasheed et al., 2019; White et al., 2020). Furthermore, it has also been indicated a potential role of vitamin C transporters in human cancer (Caprile et al., 2009; Hong et al., 2013; Aguilera et al., 2016). Particularly, genetic polymorphisms in the *SVTC2* gene have been associated to several types of tumor, including lymphoma, breast, head and neck, and gastric cancers (Ngo et al., 2019). Liu et al. (2020) showed that *GLUT3* gene expression was significantly reduced in leukemic blasts compared with normal hematopoietic cells, suggesting a defective ability in the absorption mechanisms. Furthermore, while limited data on vitamin C status are available in hematological malignancies (Aldoss et al., 2014; Nannya et al., 2014; Huijskens et al., 2016), it has been recently showed that in AML patients, plasma ascorbate levels were decreased at disease onset as opposed to healthy controls and the achievement of complete remission associated with increased values, albeit at lower-than-normal levels (Aldoss et al., 2014; Nannya et al., 2014; Huijskens et al., 2016).

## The Multifactorial Role of Ascorbic Acid Under Physiological Condition

Ascorbic acid takes part in many biochemical processes in humans, acting as an essential enzymatic cofactor, a reducing and antioxidant agent and a scavenger of ROS in biological systems. The wide spectrum of its biological functions relies on its role as a specific cofactor for the catalytic activity of the 2-oxoglutarate-dependent dioxygenases (2-OGDDs), which catalyze the addition of hydroxyl group to various substrates (Kuiper and Vissers, 2014). For instance, under physiological conditions, ascorbate is essential in the biosynthesis of collagen, the most abundant extracellular protein, promoting the proper folding of the stable collagen triple-helix conformation (Phillips et al., 1994). By increasing the extracellular matrix components, these mechanisms are supposed to prevent cancer spread, thus walling in tumors (Cameron and Rotman, 1972; Cameron et al., 1979). In addition, ascorbate may also act as a cofactor in the hydroxylation of the hypoxia inducible factor-1 α (HIF-1α), a transcription factor that regulates the expression of specific genes involved in the cellular response to hypoxia (Ivan et al., 2001; Jaakkola et al., 2001; Schofield and Ratcliffe, 2004; Ratcliffe, 2013; Simon, 2016). Under normoxic conditions, the hydroxylation of these proline residues induces proteasomal degradation of HIF-1α. In case of hypoxia, a scenarios which is very common in cancer, or in absence of ascorbate, hydroxylation is inhibited and HIF-1α is stabilized thereby initiating its downstream effects (Flashman et al., 2010). Furthermore, ascorbic acid, as a cofactor, is also involved in other important hydroxylation reactions, essential for catecholamines, L-carnitine, cholesterol and amino acids synthesis (Chatterjee et al., 1975).

Consistent with its role as a cofactor, ascorbic acid regulates DNA and histone methylation thanks to its ability to modulate 2-OGDD enzymes, which encompass demethylases involved in epigenetic regulation and in the maintenance of genomic stability (Cimmino et al., 2018). Particularly, the TET proteins are responsible for the active DNA demethylation and regulate gene transcription by converting 5-methylcytosine (5mC) to 5-hydroxymethylcytosine (5hmC), further to 5-formylcytosine (5fC) and 5-carboxylcytosine (5caC), to produce an unmethylated cytosine, thus completing the process of DNA active demethylation (Tahiliani et al., 2009; Ito et al., 2011). Ascorbic acid sustains and promotes the catalytic activity of TET enzymes, most likely reducing Fe^3+^ to Fe^2+^ (Kuiper and Vissers, 2014). Recent studies have demonstrated that ascorbic acid, as a direct regulator of TET activity, may enhance 5hmC generation, in a time- and dose-dependent fashion (Yin et al., 2013; Young et al., 2015). Two mechanisms have been proposed to explain the stimulatory effect of ascorbate on TET enzymatic activity. The first is related to its role as a cofactor, capable to directly bound the catalytic domain of TET proteins by enhancing their enzymatic activity (Hore et al., 2016); this finding is also supported by the evidence that other antioxidant compounds do not show effects on TET activity in *in vitro* models (Tahiliani et al., 2009; Ito et al., 2011; Chen et al., 2013; Dickson et al., 2013; Minor et al., 2013; Yin et al., 2013). The second hypothesis posits a different mechanism, suggesting that the stimulatory role of ascorbate on TET activity is associated to its ability to promote the reduction of Fe^3+^ to Fe^2+^ (Hore et al., 2016).

**FIGURE 2:**
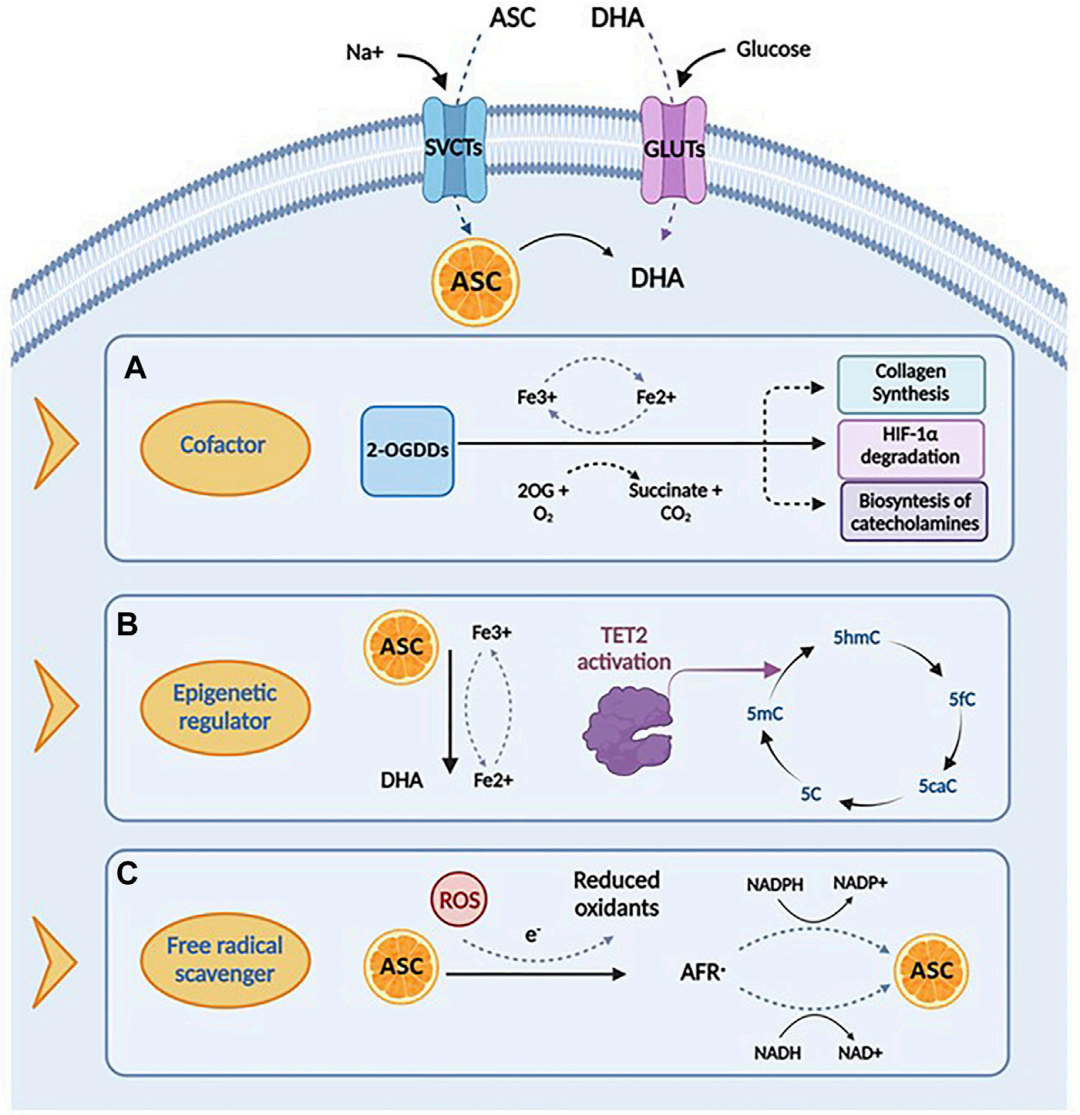
Biological functions and mechanisms of action of ascorbic acid. **(A)** Ascorbic acid plays an important role in several biological processes by acting as a cofactor for 2-oxoglutarate dependent dioxygenases (2-OGDDs) that have a wide range of biological functions, including collagen synthesis, HIF-1α degradation and biosyntesis of catecholamines. **(B)** Ascorbic acid can act as an epigenetic regulator by enanching the activity of TET2 enzyme, that catalyzes the conversion of 5-methylcytosine (5-mC), into 5-hydroxymethylcytosine (5-hmC), inducing DNA demethylation. **(C)** Ascorbic acid has important roles in scavenging free radicals, having the ability to donate an electron to reactive oxygen species (ROS) to form a relatively stable ascorbyl-free radical (AFR). Created with Biorender.com.

In addition to its function as a cofactor of several enzymes, ascorbic acid is involved in many biological processes acting as an electron donor. At physiological concentrations, ascorbate is a potent free radical scavenger, with a protective effect against oxidative damage caused by ROS, commonly produced during normal cellular metabolism (Gaziano et al., 2012). The antioxidant mechanisms of ascorbic acid are based on its capacity to reduce potentially damaging ROS and produce chemically inert resonance-stabilized ascorbate radicals (AFR). The AFR is reduced back to ascorbate intracellularly by the activity of NADH^−^ and NADPH dependent reductases, which display high affinity for the generated radicals (Duarte and Lunec, 2005; Santos-Sánchez et al., 2019). In case of accumulation of AFR in areas not accessible to these enzymes, two AFR molecules may either react with each other or dismutate to form one molecule each of ascorbate and DHA (Bielski et al., 1981). These may be then converted back into ascorbic acid for reuse or may be metabolized with further release of additional electrons (May et al., 2001; Wilson, 2002; Duarte and Lunec, 2005). This mechanism underlines the cytoprotective functions of Vitamin C, involved in the first line of antioxidant defense, including prevention of DNA mutation induced by oxidation (Noroozi et al., 1998; Pflaum et al., 1998; Lutsenko et al., 2002), protection of lipids against peroxidative damage (Barja et al., 1994; Sweetman et al., 1997), and repair of oxidized amino acid residues to maintain protein integrity (Kimura et al., 1992; Sweetman et al., 1997; Cadenas et al., 1998). The multifactorial roles of ascorbate discussed above are summarized in Figure 2.

## Ascorbate as a Key Epigenetic Regulator Involved in Hematopoietic Stem Cell Function and Leukemogenesis

As previously mentioned, among the various biological effects induced by ascorbic acid, emerging evidences suggest its key role in the epigenetic reprogramming, an effect related to the ability of ascorbic acid to act as an electron donor and, particularly, a cofactor for 2-OGDDs. These enzymes require 2-oxoglutarate (2-OG) and molecular oxygen, as substrates, and non-heme iron (Fe2+) and ascorbic acid, as cofactors; therefore, the reduction of both substrates or cofactors result in a decreased activity of these enzymes (McDonough et al., 2010; Vissers et al., 2014; Martínez-Reyes and Chandel, 2020; Crake et al., 2021). This superfamily of enzymes include key epigenetic regulators of histone demethylation and DNA hydroxymethylation, which have been shown to have crucial roles in the epigenetic regulation of stem cells and cancer (Cimmino et al., 2018). These evidences are of particular interest for AML because many of these demethylase enzymes, which require ascorbate as a cofactor, are deregulated in the process of leukemogenesis (Das et al., 2021), including lysin-specific demethylases (Castelli et al., 2018), the Jumonji C (JmjC) domain containing histone demethylases (JHDMs) (Wang et al., 2011a; Staehle et al., 2021), the ALKB homolog (ALKBH) family of nucleic acid demethylases (Gerken et al., 2007; Yi et al., 2010) and TET enzymes (Castelli et al., 2018; Das et al., 2021). Of the context discussed above, it has been recently demonstrated that ascorbic acid, acting as an epigenetic modulator could promote DNA demethylation in embryonic stem cells (ESCs), stimulate the reprogramming of fibroblast to induced pluripotent stem cells (iPSCs) and humper the aberrant self-renewal of HSCs, through the stimulation of JHDM activity (Chung et al., 2010; Esteban et al., 2010; Cimmino et al., 2018). Recent studies on JHDMs have also clearly demonstrated the involvement of these enzymes in disease initiation and progression, suggesting new attractive targets for myeloid malignancies.

Particularly, several studies have investigated the specific relationship between the activity of TET enzymes, ascorbate and the development of hematologic malignancies. It has been recently reported that ascorbic acid can protect HSCs from epigenetic alterations driving leukemia progression, stimulating the catalytic activity of TET enzymes, which are known as *bona fide* tumour suppressors of the hematopoietic lineage. Among *TET* genes, alterations in *TET2* are frequently reported in myeloid disorders, occurring in 10% of *de novo* AML, 30% of myelodysplastic syndrome (MDS) and almost 50% of chronic myelomonocytic leukemia (CMML) cases (Delhommeau et al., 2009; Papaemmanuil et al., 2016). TET2 is one of the principal epigenetic regulator of normal and malignant hematopoiesis, being able to regulate the differentiation and self-renewal of HSCs (Nakajima and Kunimoto, 2014), and is one of the most commonly mutated genes reported at high allele frequency in CD34^+^ hematopoietic stem and progenitor cells (HSPCs). These evidences suggested that *TET2* mutations are early clonal events of the leukemic transformation in cells with multi-lineage potential.

Acting as a co- factor for the 2-OGDDs, ascorbate sustains and promotes the activity of TET enzymes, as already demonstrated by *in vitro* and *in vivo* evidences (Agathocleous et al., 2017; Cimmino et al., 2017; Vissers and Das, 2018). Two recent studies provide novel insights on how ascorbic acid regulates HSC functions and leukemogenesis by the enhancement and restoration of TET2 function, respectively.

In the first, Agathocleous et al. reported that vitamin C deficiency might play a role in leukemia progression. Interestingly, the authors showed that the more immature population of stem and progenitor cells display higher levels of ascorbate than more differentiated cells. Accordingly, they found also an overexpression of the *Slc23a2* gene, which provides instructions for making a protein involved in vitamin C uptake in HSCs/MPPs (multipotent progenitors), determining an accumulation of ascorbate levels, which decreased with differentiation (Agathocleous et al., 2017). Using *Gulo*-depleted mice, which are unable to synthesize ascorbate from glucose, they showed that ascorbate depletion increased HSC pool, in part as a consequence of Tet2 reduced activity, a scenario which was reversed by dietary vitamin C supplementation. These findings suggest the beneficial role of ascorbate treatment in the setting of *TET2* mutated leukemia. Furthermore, the presence of additional internal tandem duplications (ITDs) in the juxtamembrane domain of *FLT3* cooperated with ascorbate deprivation in acceleration of leukemia development (Agathocleous et al., 2017), phenocopying *TET2* loss.

In a second, independent study Cimmino et al. established a reversible mouse model with *Tet2* knockout. *Tet2*-deficient mice showed defective self-renewal and differentiation capacity of HSC/HPC (hematopoietic progenitor cell), and defective genomic hydroxymethylation and DNA hypermethylation. Treatment with ascorbic acid pharmacologically mimicked TET2 restoration, inducing a reversal of defective DNA methylation and cell differentiation. As *TET2* mutations are almost exclusively heterozygous, ascorbic acid was found capable to stimulate the activity of the non-mutated *TET2* allele, leading to genome-wide DNA demethylation, differentiation and cell death. These evidences also suggest that the persistence of TET2 deficiency is needed to maintain leukemic self-renewal (Cimmino et al., 2017). Furthermore, the authors showed the ability of ascorbate to enhance the activity of poly(ADP ribose) polymerase (PARP) inhibitor to induce cell death, providing a safe and effective combination strategy to selectively target TET deficiency in cancer (Cimmino et al., 2017).

Altogether, these data suggest that the restoration of TET2 activity *via* ascorbate supplementation could provide an opportunity to reverse disease progression in AML cases linked to heterozygous loss-of-function mutations in *TET2*, pointing out to its role as a potentially non-toxic therapy for TET-associated malignancies (Agathocleous et al., 2017; Cimmino et al., 2017).

## The Anti-Cancer Effects of Ascorbic Acid at Pharmacologic Doses

**FIGURE 3:**
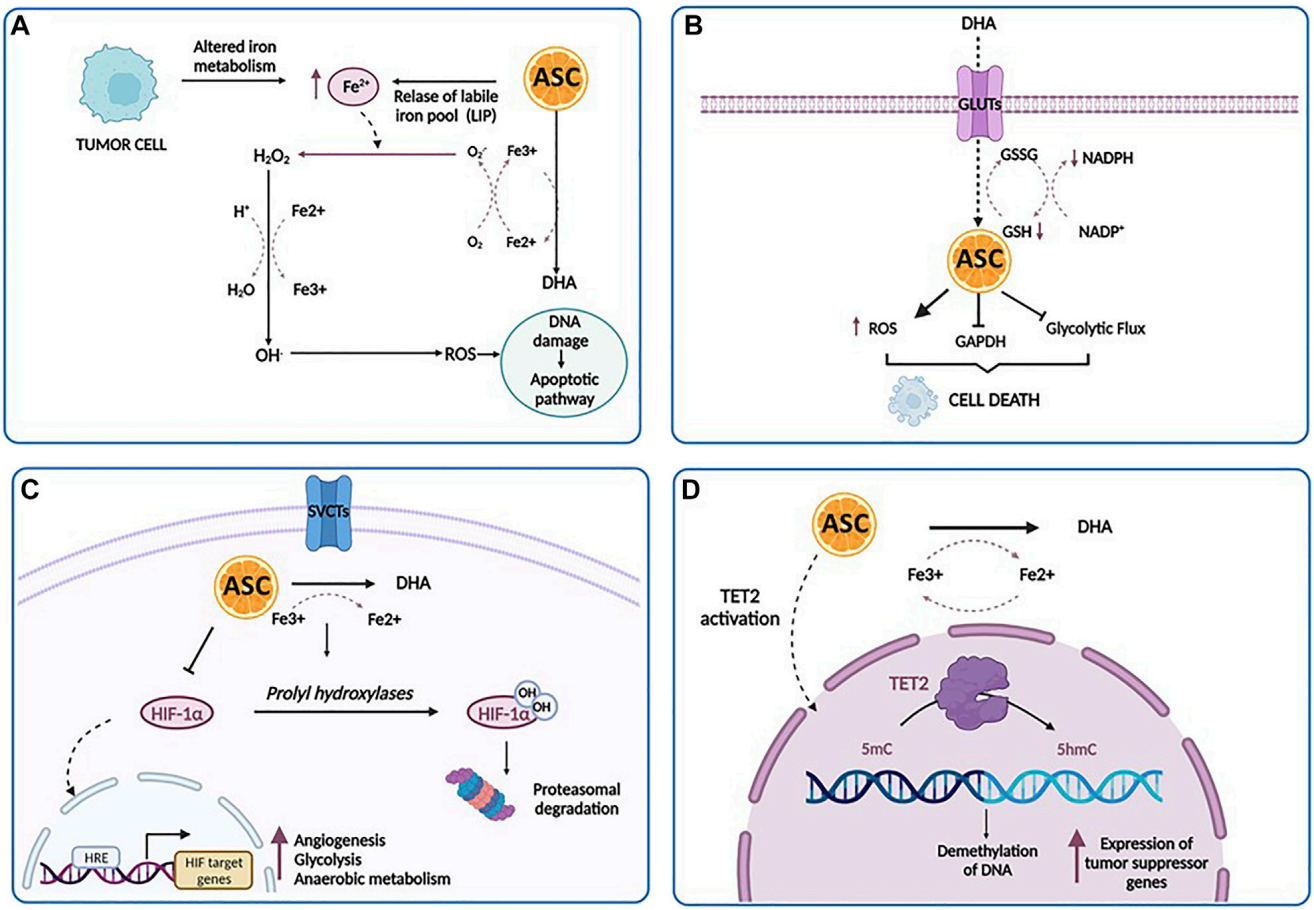
The anti-cancer effects of ascorbic acid. **(A)** A high concentration of ascorbic acid (ASC) increases the labile iron pool (LIP) of tumor cells, induces the production of increased level of ROS directly damaging mitochondria and DNA, and ultimately stimulates apoptotic pathways. **(B)** Tumor cells can uptake DHA at higher rates and then internally reduce it to ASC. This reduction triggers scavenging of glutathione (GSH), inducing oxidative stress, the inactivation of glyceraldehyde 3-phosphate dehydrogenase (GAPDH), the inhibition of glycolytic flux, an energy crisis and cell death. **(C)** In the hypoxic conditions of tumor microenvironment, there is a repression of HIF-1α hydroxilation. As a result, HIF-1α accumulates in the cytoplasm and translocates into the nucleus, promoting the transcription of its targets involved in prcesses such as angiogenesis, glycolysis and anaerobic metabolism, metastasis and resistance to therapy. Under ascorbic acid treatment, HIF-1α is hydroxylated by the prolyl hydroxylases, ultimately leading to polyubiquitination and proteasomal degratadion of HIF-1α. **(D)** Ascorbic acid binds to the catalytic domain of TET and facilitates DNA demethylation and re-expression of important tumor suppressor genes. Created with Biorender.com.

Among its many effects on cellular functions and metabolism, ascorbic acid has also shown a powerful anti-cancer effect against a number of human cell lines. Indeed, while generally regarded as an antioxidant, ascorbic acid may also have prooxidant activities at high pharmacological concentrations (Noguera et al., 2017), obtained by intravenous administration, and in the presence of free transition metal ions, especially iron. Particularly, ascorbate catalyzes the reduction of Fe^3+^ to Fe^2+^, leading to the formation of H_2_O_2_, through the so-called Fenton reaction, and exerting a cytostatic and cytotoxic effect against tumor cells, without harming normal cells. This pro-oxidant potential has been investigated in many studies in the prevention and treatment of cancers and is proposed to be dose-dependent (Putchala et al., 2013). Among the possible mechanisms, four main biological pathways have been proposed to explain how high-dose ascorbate targets several vulnerabilities of tumor cells: intracellular iron metabolism, DHA uptake *via* GLUT1, hypoxia pathways and epigenetic regulators (Figure 3).

One of the major determinants of ascorbate-mediated tumor cytotoxicity is represented by the amount and the availability of intracellular iron. The labile iron pool (LIP) represents a source of intracellular iron available for exchange between various cellular compartments (Kakhlon and Cabantchik, 2002). The intracellular iron concentration has a key role in the ascorbate-mediated cytotoxicity, related to the formation of H_2_O_2_ and .OH, which directly damages mitochondria and DNA, stimulating apoptotic pathways. Particularly, tumor cells displayed low levels of antioxidant enzymes and an impaired redox balance in respect of their normal counterpart, rendering these cells more vulnerable to oxidative stress and identifying pro-oxidant stimulations as a strategy to induce their death (Moloney and Cotter, 2018). According to these findings, Schoenfeld et al. have explored in detail the sensitivity of glioblastoma and non-small cell lung cancer (NSCLS) cell lines to ascorbate and have demonstrated that an altered iron metabolism, associated with disturbances in oxidative stress, induces an increased level of mitochondrial ROS, ultimately leading to an increase in the level of LIP and sensitivity of cancer cells to ascorbic acid (Schoenfeld et al., 2017). Interestingly, a dual relationship exists between LIP and ascorbate: pharmacological doses of ascorbate increase LIP in tumor cells, whereas an increased LIP in tumor cells enhances the toxicity of pharmacological doses of ascorbate (Du et al., 2015). Moreover, elevated LIP levels have been found in several tumor cell types as opposed to their normal counterpart, such as breast cancer cells, T-cell lymphoma and RAS-transformed cells (Yang and Stockwell, 2008; Kiessling et al., 2009; Pinnix et al., 2010). Accordingly, one could speculate that the intravenous iron injection may represent a powerful strategy to sensitize tumor cells to ascorbic acid via increased LIP levels (McCarty and Contreras, 2014).

The second mechanism involves DHA, the reversible oxidized form of ascorbic acid, based on the findings that tumor cells have the peculiar ability to uptake DHA at much higher rates and then internally reduce it to ascorbic acid (Nauman et al., 2018). As reported by Yun et al. (2015), in *KRAS* and *BRAF*-mutated colorectal cancer, this reduction triggers scavenging of glutathione (GSH), induces oxidative stress, inactivates glyceraldehyde 3-phosphate dehydrogenase (GAPDH), inhibits glycolytic flux and leads to an energy crisis with subsequent cell death (van der Reest and Gottlieb, 2016). Acting as a glycolytic inhibitor, high-dose ascorbate could represent an effective strategy against tumor cells, frequently characterized by high glycolytic activity. The third anti-tumor mechanism is based on the activity of ascorbate as a cofactor for 2-OGDDs, including HIF-hydroxylases (Ozer and Bruick, 2007). As reported above, in the hypoxic condition of tumor microenvironment, there is a repression of HIF-1α hydroxylation, associated with HIF activity. HIF-1α promotes the transition of tumor cells from aerobic to anaerobic metabolism, increasing glycolysis to maintain energy production (Masoud and Li, 2015). Thus, it is conceivable that the previously discussed DHA mechanism works in conjunction with the oxygen pathways alterations, leading to a global disruption of metabolic functioning in the tumor cell, that trigger cell death (Nauman et al., 2018). Furthermore, several studies have reported elevated levels of HIF-1α in AML, where it mediates the capacity of leukemic cells to migrate and invade extramedullary sites, suggesting that hypoxia and HIF-mediated signaling may play a crucial role in leukemia. Thus, targeting HIF with ascorbic acid could represent a potentially useful approach in AML treatment (Wang et al., 2011b; Kawada et al., 2013; Forristal et al., 2015; Gao et al., 2015).

Lastly, ascorbate modulates DNA demethylation and epigenetically reprograms cancer cells through the interaction with TET enzymes family (Blaschke et al., 2013; Ito et al., 2013). Binding to the catalytic domain, it facilitates TET-mediated DNA demethylation and re-expression of important tumor suppressor genes, with subsequent increase in chemosensitivity (Blaschke et al., 2013; Shenoy et al., 2017).

## Targeting AML Driver Mutations Using Ascorbic Acid: From *In Vitro* Experiences to Clinical Trials

Despite a growing body of evidence suggesting the anti-cancer properties of ascorbic acid, few data are available on its role for the treatment of AML.

In an *in vitro* study, Parker and coworkers demonstrated that ascorbate, at concentrations of 0.25–1 mM, was able to induce a dose- and time-dependent inhibition of proliferation in various AML cell lines (HL-60, acute promyelocytic leukemia cell line NB4, retinoic acid-resistant APL cell line NB4-R1, K562 chronic myelogenous leukemia cell line, and KG1), and primary blasts (Park et al., 2004). The induction of apoptosis in these cells was due, at least in part, to the excessive increase of H_2_O_2_ levels (Park, 2013).

Another study performed by Kawada et al. (2013) attempted to determine whether high ascorbate may exert significant cytotoxic effects against human leukemic cells, K562, HL60, Jurkat (T-lymphoblastic leukemia) and Raji (B-lymphoblastic leukemia), and normal hematopoietic cells, confirming that the specific cytotoxic effects on leukemic cells were caused by the production of H_2_O_2_ with an effect directly proportional to the dose employed. These data were confirmed *in vivo*, whereby the intravenous administration of high ascorbate repressed the proliferation of leukemic cells injected in NSG (NOD scid gamma) mice. Moreover, the authors also showed that high dose ascorbate markedly inhibited the expression of HIF-1α in leukemic cells by blocking the transcriptional activation of NF-kB, constitutively upregulated in many types of leukemia and associated with leukemic progression (Braun et al., 2006; Packham, 2008; Reikvam et al., 2009).

Accordingly, Mastrangelo et al. (2015) tested the effects of high concentrations (0.5–7 mM) of ASC on a variety of human myeloid cell lines, HL60, K562, U937, NB4, NB4-R4 and arsenic trioxide (As_2_O_3_, ATO)-resistant NB4 (NB4/As), showing the high sensitivity of myeloid leukemia cell lines to the pro-oxidant effects of high doses ascorbate, with an average cytotoxic concentration of 3 mM. Surprisingly, ASC was found significantly more effective than ATO as a single agent in inducing apoptotic cell death in HL60 human cell lines *in vitro*. Since ATO also functions as a pro-oxidant factor, in a second study, Noguera et al. (2017) tested the effects of ascorbate in combination with ATO on AML and APL primary blasts and cell lines, including NB4, NB4-R4, NB4-ATO-R, and MV4-11 [AML-M5 derived cell line with t(4;11) and *FLT3*-ITD mutations], showing a synergistic effect. Particularly, the combination treatment was highly effective in APL samples, displaying a cytotoxic mechanism linked to ROS overproduction within leukemic cells and consequent induction of apoptosis. Moreover, high concentrations of ascorbate were able to downregulate the phosphorylation of FLT3 and its downstream target proteins STAT5a/b, suggesting the potential activity of the drug also in the subset of *FLT3*-ITD positive AML. In an attempt to further characterize the mechanistic underpinnings underlying the efficacy of ascorbic acid in the APL setting, the same authors found that the fusion protein PML/RARα inhibits NRF2 (NF-E2 p45-related factor 2) functions. This is a transcription factor that orchestrates cellular adaptive responses to stress, and whose nuclear transfer is prevented by ascorbate treatment, thereby enhancing its degradation into the cytoplasm. As a result, the inhibition of the NRF2 oxidative stress pathway clarifies the peculiar sensitivity of APL cells to the pro-oxidant activity of high-dose ascorbate and suggest its potential use in APL patients, especially in those resistant to ATO/retinoic acid treatment (Banella et al., 2019). Moreover, ascorbate also shows the ability to induce cell death by targeting glycolytic metabolism in primary AML blasts, through the inhibition of hexokinase 1/2 (HK1/2) and GLUT1 in hematopoietic cells, and, in combination with the metabolic inhibitor buformin, also decreases mitochondrial respiration and ATP production, sparing healthy CD34^+^ cells. Overall, these data clearly depict an effect of ascorbate on glycolysis and contribute to elucidate the targets and mechanisms through which this therapeutic agent exerts its anti-cancer effects (Banella et al., 2022).

Another study investigated instead the combinatorial effect in AML cells of low-dose of ascorbate with decitabine, an HMA widely used in the treatment of AML in elderly patients, not suitable for conventional intensive chemotherapy. Potentially, one of the mechanisms for the efficacy of decitabine treatment is the upregulation of TET2 proteins, among others. Acting as a direct regulator of TET activity, ascorbate increases 5hmc levels and potentially sensitizes patients to decitabine. Indeed, *in vitro* studies showed that this combination in NB4 and HL60 cells resulted in the most significant upregulation of the activity of TET2 enzyme. Furthermore, *in vivo* results displayed a synergistic effect of this two agents, which are able to improve the complete remission (CR) rate in elderly AML patients (Zhao et al., 2018). Overall, these observations are of potential interest particularly for MDS and AML patients, who could have an additional benefit from adding ascorbic acid to HMA therapy.

**FIGURE 4:**
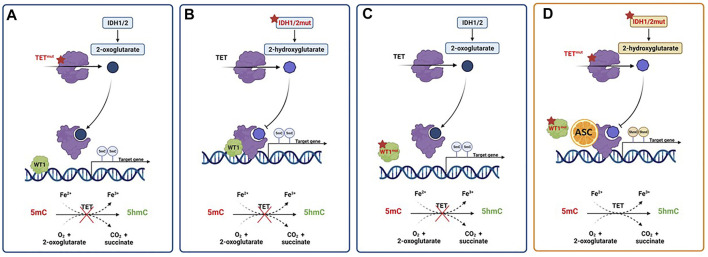
Activity of pharmacologic doses of ascorbic acid in AML with TET2, IDH1/2 or WT1 altered pathways. **(A)** TET2 mutations result in a nonfunctional enzyme with hypermethylation of gene promoters, and prevention of oxidation of 5-methylcytosine (5mC) to 5-hydroxymethylcytosine (5hmc). **(B)** The presence of gain-of-function mutations in IDH1/2 genes results in the overproduction of the oncometabolite 2-hydroxyglutarate (2-HG) with the inhibition of TET2 activity. **(C)** WT1 mutations hamper the ability of TET2 to bind and activate WT1, inhibiting the expression of WT1-target genes. **(D)** Ascorbic acid treatment mimics TET2 restoration, inducing a reversal of defective DNA methylation and cell differentiation, ultimately inhibiting tumor progression. Created with Biorender.com.

Interestingly, ascorbate specifically interacts with the C-terminus catalytic domain of TET2, inducing effects not shown with other strong reducing chemicals (Yin et al., 2013). As already mentioned, ascorbate can act as an epigenetic therapeutic in the presence of heterozygous *TET2* mutations by restoring TET2 activity and providing an opportunity for reversing disease progression in AML cases linked to heterozygous loss-of-function mutations in *TET2*. Thus, the proposed mechanism of action requires the presence of residual functional enzyme (Das et al., 2021). However, TET2 mutations frequently occur in combination with other lesions, but little is known about how the genomic makeup may impact the up-regulation of TET2 activity induced by ascorbic acid as well as its subsequent effects on cell differentiation and survival (Papaemmanuil et al., 2016; Das et al., 2021). To further muddy the waters of the intertwining relation between TET enzymes and ascorbate in myeloid disorders, a reduced TET2 activity may also result from mutations in *IDH1*, *IDH2* and *WT1*, frequently reported in AML (Figure 4). In the presence of such mutations, IDH enzymes produce 2-hydroxyglutarate (2-HG), an oncometabolite able to acts as a competitor for TET2, instead of its physiological substrate, 2-oxoglutarate (2-OG) (Figueroa et al., 2010), causing functional inactivation of TET2 enzyme. Moreover, the presence of mutated WT1, prevent the recruitment of TET2 to DNA and the activation of the WT1-target genes expression. These findings provide the evidence of decreased TET2 activity in both context of *IDH* and *WT1* mutations, and are in line with a recent study reporting TET2 deficiency in up to 74% of patients with myeloid disorders, a result that goes beyond the mere presence of *TET2* mutations. Indeed, loss of TET2 functions by mutations or down-modulation due to various mechanisms have been identified as a common lynchpin of myeloid malignancies, as also indicated by the meta-analysis performed in the above-mentioned study (Gurnari et al., 2022). The implications of this are obvious, and suggest the potential application of ascorbate in settings beyond the disruption of TET2-IDH-WT1 pathway, which is the scenario where we would expect its maximal therapeutic efficacy, as also demonstrated by anecdotal case reports (Das et al., 2019). Furthermore, TET3 upregulation has also been invoked as a potential mechanism compensating the general TET2opathy of myeloid disorders, and has been linked to better survival outcomes in MDS with TET2 deficiency. Perhaps, ascorbate treatment may rescue the fraction of patients not able to contra-balance TET2 loss, thereby improving their dismal clinical outcomes (Gurnari et al., 2021, 2022).

As said, IDH1/2 mutations alter the epigenome of AML cells. Mingay and coworkers explored the effect of ascorbic acid in the setting of a murine leukemic model expressing *IDH1* ^R132H^ mutation. Ascorbate treatment induced a reduction in cell proliferation and an increased expression of genes involved in leukocyte differentiation in *IDH1* ^R132H^ mice, not observed in *IDH1* ^wt^ counterparts. These marked effects on cell differentiation were related to the induction of demethylation at the level of DNA binding sites of the hematopoietic transcription factors CEBPβ, HIF-1α, RUNX1 and PU.1 (Mingay et al., 2018). The previously mentioned study by Cimmino et al. (2017) provided the evidence that ascorbic acid treatment is able to induce the restoration of TET2 function in various leukemia models, by blocking aberrant self-renewal and leukemia progression. Indeed, mimicking TET2 restoration ascorbate treatment suppresses leukemic colony formation and leukemic progression of primary human leukemia patient-derived xenografts (PDX). Finally, a recent study investigated the effects of ascorbate on cell growth and differentiation of SKM-1 AML cell line, harbouring both *TET2* and *TP53* mutations, showing a beneficial anti-proliferative effect also in this subgroup of adverse-risk AML (Smith-Díaz et al., 2021).

Taken together these results identify ascorbic acid as a novel metabolic tumor suppressor involved in epigenetic remodeling and highlighted that supra-physiological doses could prevent myeloid disease progression, pointing out to its role as a potentially non-toxic therapy, especially for TET-deficient malignancies (Agathocleous et al., 2017; Cimmino et al., 2017; Guan et al., 2020). These observations suggest the incorporation of high-dose ascorbate as an adjuvant to standard chemotherapy or HMA therapy in clinical trials. Indeed, many clinical trials (NCT03682029, NCT03999723) are currently investigating the effects of ascorbate alone or as an add-on to classic therapeutic schemes of AML and myeloid disorders. Beyond HMA, specific lines of research are exploring the possibility of combining ascorbate with class I/II histone deacetylases (HDAC) (Zhang et al., 2017) or sirtuin activators (Sun et al., 2018), with the rationale of regulating TET dioxygenase-dependent effects of vitamin C (Guan et al., 2021). A peculiar application currently under investigation is the scenario of clonal hematopoiesis of indeterminate potential (CHIP) or its age-related counterpart ARCH (age-related clonal hematopoiesis), especially sustained by *TET2* mutations, where the consideration of a simple, over-the-counter supplementation of high-dose of vitamin C may represent an appealing option in decelerating progression to overt, fully-blown myeloid neoplasms (MDS/AML) (Miller and Steensma, 2020). To this end, a recent study showed that elderly individuals with inadequate vitamin C dietary intakes and plasma concentrations had higher odds of ARCH, typically TET2-related (Chen et al., 2021).

By this virtue, ascorbate could provide therapeutic opportunities able to overcome the TET2 impairment typical of myeloid neoplasms by re-establishing the net, residual TET-dioxygenase activity. Indeed, it is known that when TET2 expression is downregulated, two other dioxygenases (TET1, and especially TET3) maintain a minimal enzymatic activity critical for cell survival (Gurnari et al., 2022). Given the current evidence, newer approaches of personalized medicine should take into account not only cytogenetic and mutational characteristics but also both transcriptomic changes (e.g., RNA-seq) and assessment of vitamin C levels in patients at AML onset. This information would enable the identification of therapeutic vulnerabilities in individual patients (e.g., ascorbate in TET2-IDH-WT1 impairment and/or vitamin C-deficient patients). Ideally, with the use of sophisticated methods of artificial intelligence, one could speculate that the *in silico* creation of “digital twins” would allow testing of multiple, combinatorial therapeutic strategies (including ascorbate, conventional cytotoxic, HMA or new targeted agents), ultimately providing the best drugs to combine with this agent in a “synthetic lethally” fashion (Björnsson et al., 2019).

That said, the exact position of ascorbate in the therapeutic arsenal of AML is yet to be clearly defined. Future data derived from ongoing clinical trials will shed light on its role within the treatment algorithm of AML, a disease where still only less than 30% of patients become long-term survivors and for which new treatment options are urgently needed.

## Conclusion

The therapeutic potential of ascorbic acid in leukemia have been known for several decades. In particular, the observation that leukemic patients display low vitamin C plasma levels, due to the increased uptake by the actively proliferating leukocytes (Stephens and Hawley, 1936; Kyhos et al., 1945; Barkhan and Howard, 1958) suggested the rationale for the use of high-doses of ascorbic acid, not only as a prophylactic measure, but also to treat a number of pathologic conditions, including cancer (Pauling, 1980). Its role as an anti-cancer agent has long been debated and the identification of potential mechanisms through which ascorbate exerts its biologic and pharmacologic activities, lead to hypothesize a new window of therapeutic opportunities.

Recent investigations highlighted the role of ascorbic acid as a critical regulator of cellular epigenetic processes, and a potential drug in the therapeutic armamentarium of acute myeloid leukemia through its stimulatory effect on TET2 function. Being epigenetic dysregulation a hallmark of AML and playing such a central role in the initiation and maintenance of the disease, the possibility to overcome the dysregulated gene-expression programs through an ascorbate based-epigenetic therapy represents a promising and cost-effective anti-leukemia approach.

Overall, these findings suggest the clinical benefit that could derive from the use of ascorbic acid both as a dietary supplement or as a therapeutic agent. Despite the wide number of *in vitro* experiments demonstrates the anti-leukemia activity of ascorbic acid, the lack of robust evidences about the precise mechanism of action, the tolerability and timing of pharmacological doses of ascorbic acid *in vivo* did not allow to design appropriate clinical trials. Future clinical trials are warranted to identify patients and specific AML subgroups who may benefit the most from this therapeutic strategy.
